# Role and mechanism of Glut-1 and H+/K+-ATPase expression in pepsin-induced development of vocal cord leukoplakia

**DOI:** 10.1007/s00405-021-07172-y

**Published:** 2021-11-20

**Authors:** Yin-Jie Ao, Ting-Ting Wu, Zai-Zai Cao, Shui-Hong Zhou, Yang-Yang Bao, Li-Fang Shen

**Affiliations:** Department of Otolaryngology, The First Affiliated Hospital, College of Medicine, Zhejiang University, 79 Qingchun Road, Hangzhou City, 310003 People’s Republic of China

**Keywords:** Vocal cord leukoplakia, Precancerous lesion, Laryngopharyngeal reflux, Pepsin, Glut-1, H^+^/K^+^-ATPase

## Abstract

**Purpose:**

We investigated the role of Glut-1 and H^+^/K^+^-ATPase expression in pepsin-induced development of human vocal cord leukoplakia cells (HVCLCs). Next, we analyzed the relationship between Glut-1 and H^+^/K^+^-ATPase expression with the clinicopathological features of laryngeal carcinoma.

**Methods:**

Glut-1 and H^+^/K^+^-ATPase expression levels in HVCLCs were determined after treatment with artificial gastric juice containing pepsin and laryngeal carcinoma tissues.

**Results:**

Exposure to pepsin-containing artificial gastric juice significantly enhanced the migration and proliferation of VSCLCs in a time-dependent manner. The apoptotic rate of VSCLCs decreased over time after exposure to pepsin and reached a nadir on day 7 (*p* < 0.01). With increasing duration of exposure to pepsin, the proportion of VSCLCs in G0/G1 phase decreased and the proportions in the S and G2/M phases significantly increased (*p* < 0.05). After treatment with pepsin-containing artificial gastric juice, RT-PCR and Western blotting showed that the expression of Glut-1 and H^+^/K^+^-ATPase α, β significantly increased in HVCLCs compared to in the absence of pepsin (*p* < 0.05). The expression of Glut-1 and H^+^/K^+^-ATPase α, β gradually increased from vocal cord leukoplakia (VLC) to laryngeal carcinoma (*p* < 0.05). Lentivirus-mediated inhibition of Glut-1 expression in VCL significantly inhibited the cells’ migration and proliferation (*p* < 0.05) but enhanced their apoptosis (*p* < 0.05). Also, inhibition of Glut-1 expression resulted in an increased proportion of cells in G0/G1 phase and a significantly decreased proportion in G2/M phase (*p* < 0.05).

**Conclusions:**

Elevated Glut-1 expression may promote the development of VCL by upregulating laryngeal H^+^/K^+^-ATPase expression to reactivate absorbed pepsin, thus damaging the laryngeal mucosa.

**Supplementary Information:**

The online version contains supplementary material available at 10.1007/s00405-021-07172-y.

## Introduction

Vocal cord leukoplakia (VCL), which is a clinical diagnosis with an underlying pathology characterized by various lesions (*e*.*g*., squamous hyperplasia with or without epithelial keratosis, as well as vocal cord mucosal dysplasia), is a common laryngeal precancerous lesion [1]. Its malignant transformation rate varies from 2 to 74% [2, 3]. The underlying etiologies of VCL are long-term smoking and alcohol abuse, inhaled irritant substances, and viral infection such as HPV [4]. Research has focused on the role of laryngopharyngeal reflux (LPR) in the development of VCL. LPR refers to the reflux of gastric contents above the upper esophageal sphincter, which causes a series of symptoms and signs [5].

Gastric contents include gastric acid and non-acid components such as pepsin, cholate, bacteria, and trypsin [6]. VCL is associated with LPR [1, 4]. Weakly acidic LPR plays an important role in the pathological changes seen in VCL. We reported high expression of pepsin in vocal cord polyp and VCL, and pepsin expression in VCL significantly increased according to the grade of dysplasia [1]. However, the role and mechanism of pepsin in the development of VCL are unclear.

Pepsin can be secreted by only the chief cells of the stomach. Pepsin may reach the larynx by adhering to laryngeal cell membranes, or through absorption by laryngeal epithelial cells via receptor-mediated endocytosis at pH 7, followed by storage in vesicles and transport to intracellular vesicles such as Golgi bodies [7–9]. Pepsin under these conditions is stable but inactivated or dormant. Pepsin is reactivated by the reduction in the pH of the laryngeal microenvironment [4, 7–10]. Tissue damage caused by pepsin activity may be mediated by the epithelial-mesenchymal transition (EMT), cellular mitochondria [11–13], oxidative DNA damage [14], and laryngeal mucosal protective proteins [15].

H + /K + -ATPase proton pumps have been found in a number of extra-gastric tissues, including the larynx [16–23]. H + /K + -ATPase was found in normal laryngeal tissues, and its expression was higher in laryngeal carcinoma [19]. The function of gastric H + /K + -ATPase is gastric acid secretion. It is speculated that extra-gastric H + /K + -ATPase proton pumps also secrete acid. Therefore, we suggest that the acid secreted by H + /K + -ATPase in the larynx decreases the pH, reactivating pepsin in the laryngeal mucosa and damaging laryngeal epithelial cells.

Chronic pepsin stimulation may result in further laryngeal epithelial damage and transformation of VCL, which is a long-term process involving multiple factors. Glucose metabolism involves a metabolic shift from oxidative phosphorylation (OXPHOS) to glycolysis [24, 25]. Glucose transporter-1 (Glut-1) is an important regulator of energy demand during the development of precancerous lesion cells [26, 27]. High expression of Glut-1 enhanced glycolysis, increasing lactate28 and reducing the microenvironment pH. The decreased pH may reactivate pepsin. Therefore, we suggest that Glut-1 is key in pepsin-induced development of VCL.

To test the above hypotheses, we first investigated the expression and role of Glut-1 and H^+^/K^+^-ATPase in the proliferation, growth, and development of human vocal cord leukoplakia cells (HVCLCs) in pepsin containing artificial gastric contents. Next, we verified the expression of Glut-1 and H^+^/K^+^-ATPase in VCL and laryngeal carcinoma tissues and analyzed the relationship between Glut-1 and H^+^/K^+^-ATPase expression with the clinicopathological features of laryngeal carcinoma.

## Materials and methods

This study was approved by the institutional review board of The First Affiliated Hospital, College of Medicine, Zhejiang University, China (No. 201901022). All authors had access to the study data and reviewed and approved the final manuscript, and the study was conducted in compliance with the Declaration of Helsinki.

### Primary culture of HVCLCs

#### Clinical information

A 68-year-old man presented with a 2-month history of sustained hoarseness, sore throat, and dysphagia. His medical history included 13 years of hypertension, which was controlled by Amlodipine. Suspension laryngoscopy showed that his bilateral vocal cords were covered by gray-white keratinized tissue. The pathological results (No. F201710918) confirmed the clinical diagnosis of leukoplakia of the vocal cords. Specifically, small pieces of squamous epithelium indicated hyperplasia, and the differentiation was good.

#### Primary cell culture

Fresh leukoplakia tissue obtained during surgery was rapidly transferred into cold double-anti-Dulbecco’s modified Eagle’s medium (DMEM)/F12, transported to the laboratory and processed immediately. The tissue block was rinsed three times with sterile D-Hank’s solution and placed in a sterile vial. Next, 2.4 mg/mL Dispase II solution was added to digest the tissue. The tissue block was removed, and ophthalmic forceps were used to separate the epithelial tissue from the underlying connective tissue. The epithelial tissue was rinsed two or three times with D-Hank’s solution.

#### Cell harvesting

Configured digestive solution (0.25% trypsin and 0.02% ethylenediaminetetraacetic acid; 1:1), was prewarmed in an incubator. Epithelial tissue was cut into small pieces (0.5–1 mm^3^), which were immersed in the digestive solution and incubated at 37 °C for 20 min. After centrifugation (1000 rpm, 5 min), the supernatant was discarded, and the tissue was washed three times in Hank’s solution. The process was repeated twice. The cells were suspended in complete medium and passed through a 100-μm-mesh filter. The remaining cells were suspended in DMEM with 1% penicillin/streptomycin and 10% fetal bovine serum. A 25-cm^3^ disposable culture flask was seeded with 5 × 10^5^ cells and incubated for 1 h at 37 °C in a humidified atmosphere containing 5% CO_2_. The medium was changed every 2 days and the condition of the cells was observed daily under a phase-contrast microscope.

#### Cell Counting Kit-8 assay

Cell Counting Kit-8 (CCK-8) solution (50 μL) was added to the dishes and cultured for 4 h at 37 °C. Pipetted 100 μL per well and transferred them to specific 96-well plate. The absorbance at 450 nm was determined using an enzyme-linked immunosorbent assay reader.

#### Immunofluorescence of vimentin, CEA, and keratin

Supernatant was aspirated and the dishes were washed twice with phosphate-buffered saline (PBS). Cells in the experimental groups were fixed in 4% paraformaldehyde at 4 °C overnight, washed in PBS three times for 5 min each, and incubated with 0.5% Triton X-100 (in PBS) for 10 min. Next, the cells were washed in PBS for 5 min, blocked with 1% bovine serum albumin for 30 min, and cultured in the indicated 1:50-diluted primary antibody solution at 4 °C overnight. The cells were washed in PBS three times for 5 min each and cultured in diluted secondary antibody solution at 37 °C for 2 h. The cells were washed in PBS three times for 5 min each and stained with 4',6-diamidino-2-phenylindole for 3 min. Finally, the cells were washed in PBS three times for 5 min each, sealed in 50% glycerol, and visualized and photographed by laser confocal microscopy.

#### Three-dimensional culture of HVCLCs, laryngeal carcinoma Tu212 cells and AMC-HN-8 cells

Leukoplakia epithelial cells, laryngeal carcinoma Tu212 cells (Icell Bioscience, Shanghai, China), and AMC-HN-8 cells (BnBio, Beijing, China) were suspended at 1 × 10^8^ cells/mL. Matrigel was stored at  – 20 °C, thawed at 4 °C for 1 day, and 1 mL was placed on ice. Matrigel (1 mL) was mixed with 1 mL of DMEM, and 1 mL of cell suspension was mixed with 2 mL of Matrigel-DMEM. The above processes were carried out on ice. The cell suspension (0.6 mL) was added to five wells of a 24-well plate, which was transferred to a 37 °C cell-culture incubator for 12 h. Next, 1 mL of DMEM was added to each well and the plate was returned to the incubator. The upper liquid medium was replaced every other day, and cell growth was monitored daily under a light microscope.

#### Exposure of HVCLCs to artificial gastric juice

Artificial gastric juice (Soleibao, Beijing, China) comprised 8.2 mL of 9.5–10.5% HCL + 400 mL diluted 0.03 mol/L NaCl, to which was added 5 g pepsin; the final concentration was 0.01 g/mL, pH 2. The experimental groups were: Group 1, HVCLCs without artificial gastric juice treatment (two-dimensional [2D] 0, three-dimensional [3D] 0, respectively). Group 2, artificial gastric juice treatment for HVCLCs for 3 consecutive days (2D 3, 3D 3). Group 3, artificial gastric juice treatment for HVCLCs for 5 consecutive days (2D 5, 3D 5). Group 4, artificial gastric juice treatment for HVCLCs for 7 consecutive days (2D 7, 3D 7). Artificial gastric juice (1500 µL) was added once a day.

#### Transfection of HVCLCs with Glut-1 low-expressing lentivirus

HVCLCs were transfected with Glut-1 low-expressing lentivirus (HanBio, Shanghai, China). Briefly, 1 day before virus transfection, a certain number of cells were transferred to a culture dish. When cells reached about 50% confluence, they were transfected. The cells were placed in a 37 °C incubator for 20 h. The virus was dissolved in an ice bath and pipetted gently for mixing. The lentivirus titer was 1 × 10^8^ TU/mL, and the MOI was 15. The primary culture medium was aspirated, virus-containing culture medium was added, and the dish shaken to ensure that medium covered the cells. The cells were incubated at 37 °C overnight. One day after transfection, the virus-containing medium was discarded, and fresh DMEM was added. The cells were incubated at 37 °C. The success of viral transfection was determined by fluorescence excitation. When the virus transfection rate reached about 80%, antibiotics were added to screen the cells. Finally, the cells were divided into three groups: HVCLCs transfected with Glut-1 low-expressing lentivirus, HVCLCs and HVCLCs carries only the fluorescent framework were used as controls.

#### Transwell assay

Cells were digested with trypsin, centrifuged, and enumerated. The cell density was adjusted to 5 × 10^5^ /mL. Matrigel was diluted with DMEM at a ratio of 8:1. Diluted Matrigel (40 µL) was added to the Transwell chamber and cell suspension (150 µL) was added to the Transwell compartment. DMEM (800 µL) containing 20% FBS was added to the lower chamber. The Transwell chamber was removed, and the culture medium in the six-well plate was discarded. The Transwell chamber was washed with PBS, fixed in 4% formaldehyde for 20 min, washed with PBS for 5 min, and stained with 0.1% crystal violet for 30 min. The upper layer of unmigrated cells was removed with cotton swabs, and the Matrigel was removed. The cells were enumerated and photographed. Each experiment was performed in triplicate.

#### Wound-healing assay

A two-well cell-scratch module was used to ensure a scratch width of 500 µm. The module was placed in a 24-well plate and 70 µL of a cell suspension (5 × 10^5^/mL) were added to both sides of the module. After 6 h, the cells were confirmed to be completely attached to the wall. Sterile forceps were used to remove the module, and 1 mL of culture medium was added along the plate wall. Finally, healing of the scratches was monitored visually.

#### CCK-8 assay

Cell suspension (100 μL) was added to a 96-well plate and 100 μL of DMEM was added to each well. Next, 10 μL of CCK-8 was added to each well. The cells were incubated at 37 °C for 2 h. The absorbance of each well at 450 nm was determined using the Spectra Plus Microplate Reader (Molecular Devices, Sunnyvale, CA).

#### Flow cytometry

After digestion, cell suspension was dripped into a centrifuge tube containing anhydrous alcohol and PBS (3:1) and fixed for 24 h. The cells were next washed with PBS, transferred to a tube, centrifuged (1000 rpm, 5 min), and the supernatant discarded. Cell-cycle-staining buffer was added, and the tube was placed in the dark for 30 min. Cells (5 × 10^5^) were collected and centrifuged (1000 rpm, 5 min). The medium was discarded, the cells were washed with 3 mL of PBS, passed through a 400-mesh filter, and centrifuged (1000 rpm, 5 min). Next, the cells were placed in the dark at 4 °C for 30 min and the proportions undergoing non-apoptotic programmed cell death and apoptosis, and the proportions in the various stages of the cell cycle, were evaluated by flow cytometry in triplicate and analyzed by CytExpert software (Beckman Coulter, Inc., CA).

### Patients and tissue samples

We analyzed 30 pathological specimens from patients with laryngeal carcinoma, 30 from patients with VCL, and 30 samples of paracarcinoma tissue (taken 0.5 cm from the negative margin after tumor excision) from patients with laryngeal carcinoma treated at our hospital between November 2014 and July 2020. The inclusion criteria were: for the leukoplakia group, a confirmed pathological diagnosis of squamous epithelial hyperplasia or mild, moderate, or severe atypical hyperplasia; for the laryngeal carcinoma group, a confirmed pathological diagnosis of squamous cell carcinoma. Exclusion criteria were preoperative radiotherapy and/or chemotherapy and/or immunotherapy; treatment with PPIs; coexisting metabolic or autoimmune diseases; incomplete medical records; and/or loss to follow-up.

### Quantitative real-time RT-PCR

Total RNA was isolated according to the manufacturer’s instructions. Briefly, 1 µg of RNA was reverse-transcribed using a First-Strand cDNA Kit (EZB-RT2GQ SYBR ON) and subjected to PCR using a SYBR Green qPCR Kit (A0001-R2 SYBR ON), incubated at 42℃ for 15 min, and stored at  – 20 °C. RNA primers were designed and synthesized by Sangon. The primers for Glut-1 (Abcam, Cambridge, UK) were forward 5'-GGTCATGAGTATGGCACAACC-3' and reverse 5'-GTCAACACGGCCTTCAC-3'. The primers for H^+^/K^+^-ATPase α (Abcam) were forward 5'-CATCATCGCCAGCTTTAAGAAC-3' and reverse 5'-CAGCGTTGATCTGGAATTTGTC-3'. The primers for H^+^/K^+^-ATPase β (Abcam, Cambridge, UK) were forward 5'-CAGTCTGCACTACTTCCCTTAT-3' and reverse 5'-CACTTTCCCTTCATACGGGTC-3'. The primers for anti-glyceraldehyde 3-phosphate dehydrogenase (GAPDH) (CST, Boston, MA) were forward 5'-GAAGGTGAAGGTCGGAGTC-3' and reverse 5'-GAAGATGGTGATGGGATTTC-3'. The PCR products were 111 bp (Glut-1), 80 bp (α-subunit), 175 bp (β-subunit) and 172 bp (GAPDH) in length. The 2^ΔΔCt^ method was used to calculate relative gene expression levels. The experiments were performed in triplicate.

### Western blotting

Western blotting was performed in accordance with the manufacturer’s instructions. Rabbit monoclonal anti-Glut-1 (1:1000),  – H^+^/K^+^-ATPase α (1:2,000), and  – H^+^/K^+^-ATPase β (1:1,000) were purchased from Abcam (Cambridge, UK), and GAPDH (1:1,000) from CST (Boston, MA). The secondary antibodies were goat anti-rabbit antibodies (1:1,000) conjugated with horseradish peroxidase (Abcam). Signals were visualized using ImageJ software (National Institutes of Health, Bethesda, MD). Protein levels were quantified by scanning densitometry in triplicate.

### Immunohistochemistry

Paraffin sections were cut at 5 µm thickness. After deparaffinization and hydration, the sections underwent antigen retrieval by the microwave-oven method. Endogenous peroxidase activity was blocked in 3% H_2_O_2_ for 25 min at room temperature. Next, the slides were incubated with primary antibodies against Glut-1 (1:200), H^+^/K^+^-ATPase α (1:200), and H^+^/K^+^-ATPase β (1:200) overnight. The next day, the sections were incubated with the corresponding secondary antibodies (1:200) at room temperature for 50 min, stained using a DAB Staining Kit and subjected to hematoxylin-and-eosin staining. The sections were photographed under a microscope; cells labeled with brownish-yellow granules were considered positive. Five high-magnification fields (× 400) were randomly selected, in each of which 100 cells were counted; scoring was as follows: 0; 1, < 25%; 2, 26–50%; and 3, ≥ 50% positive cells. Dye depth was scored as follows: 0, no staining; 1, light yellow; 2, brownish-yellow; and 3, deep brownish-yellow. Immunohistochemical data were assessed as the positive-cell score + the dye-depth score. Total scores of 0–1, 2, 3–4, and 5–6 were considered negative (–), weakly positive ( +), positive (+ +), and strongly positive (+ + +), respectively. The investigator was blinded to the group allocation.

### Statistical analysis

Associations of Glut-1 and H^+^/K^+^-ATPase expression with clinicopathological parameters were assessed by the chi-squared test or Fisher exact test. Continuous data are expressed as means ± standard deviations and were analyzed by dependent *t*-test for within-subject differences. A Pearson correlation analysis was conducted. *P*-values < 0.05 were indicative of statistical significance. GraphPad Prism 7 software was used for graphing and statistical analysis was performed using SPSS Statistics for Windows (ver. 19.0; IBM Corp., Armonk, NY).

## Results

### Role of Glut-1 and H + /K + -ATPase α, β in pepsin-induced development of vocal cord leukoplakia

#### Two- and three-dimensional culture of primary vocal cord leukoplakia cells

**Fig. 1:**
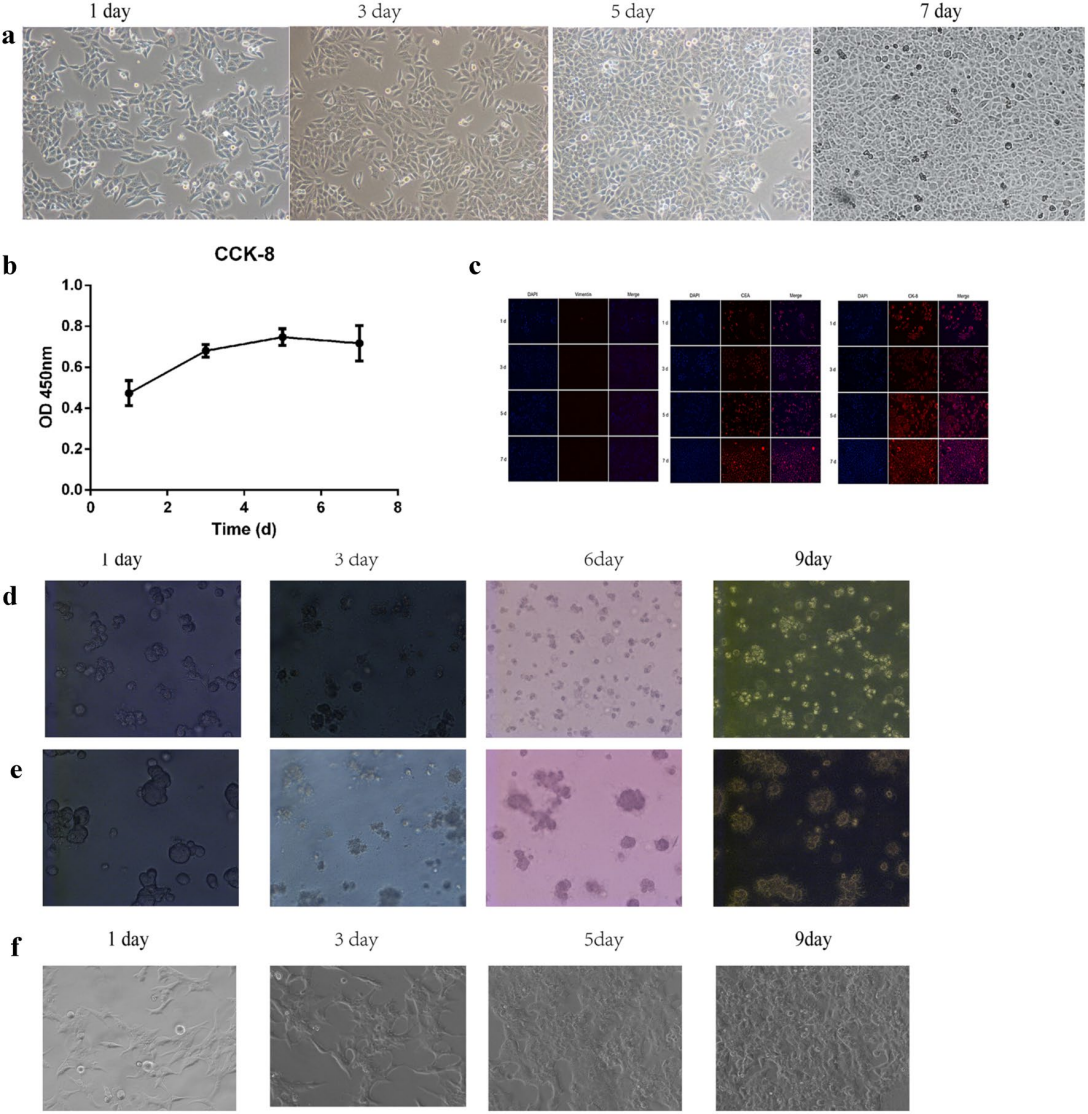
Culture of VCL cells in 2D and 3D. **a** Morphology of third-generation HVCLCs 2D cultured for 1, 3, 5, and 7 days. **b** CCK-8 assay. **c** Expression of vimentin, CEA, and CK-8 by immunofluorescence. **d** 3D culture of HVCLCs for 1, 3, 6, and 9 days. 3D model of (**e**) laryngeal carcinoma Tu212 cells and (**f**) AMC-HN-8 laryngeal carcinoma cells

VCL cells grew densely at the bottom of the culture dish. The cells were spindle-shaped and closely packed, reminiscent of paving stones. The cells were fused, elongated, transparent, and rich in cytoplasm, and the nucleus was located at the center. On day 7 after passage, their growth state was good, and the cells were passaged daily thereafter (Fig. 1a). The cells ceased proliferating after 5 days; the mean doubling time was 2–3 days (Fig. 1b). To identify leukoplakia epithelial cells, we performed immunofluorescence for CEA and CK-8, markers of leukoplakia epithelial cells. Of the cells, > 90% were positive for CEA and CK-8 but < 5% were positive for vimentin, in 2D culture (Fig. 1c). These characteristics are in accordance with those of leukoplakia epithelial cells.

By comparison, fewer spindle-shaped cells were observed in 3D culture. In addition, leukoplakia epithelial cells were stratified and distributed mainly at the gel-liquid interface. On day 3, mature leukoplakia epithelial cells of typical paving-stone arrangement were observed. Cell clusters started to appear, with ever-increasing 3D profiles. On day 6, large cell clusters were interconnected by pseudopods. On day 9, Matrigel was occupied by cells (Fig. 1d). On day 1, several spindle-shaped laryngeal carcinoma Tu212 and AMC-HN-8 cells were observed, and several small colonies were evident on day 3. On day 6, a number of spindle-cell colonies were present and on day 9, many cell colonies were visible (Fig. 1e, f).

### Pepsin modulates the migration, proliferation, apoptosis, and cell-cycle distribution of VSCLCs

**Fig. 2:**
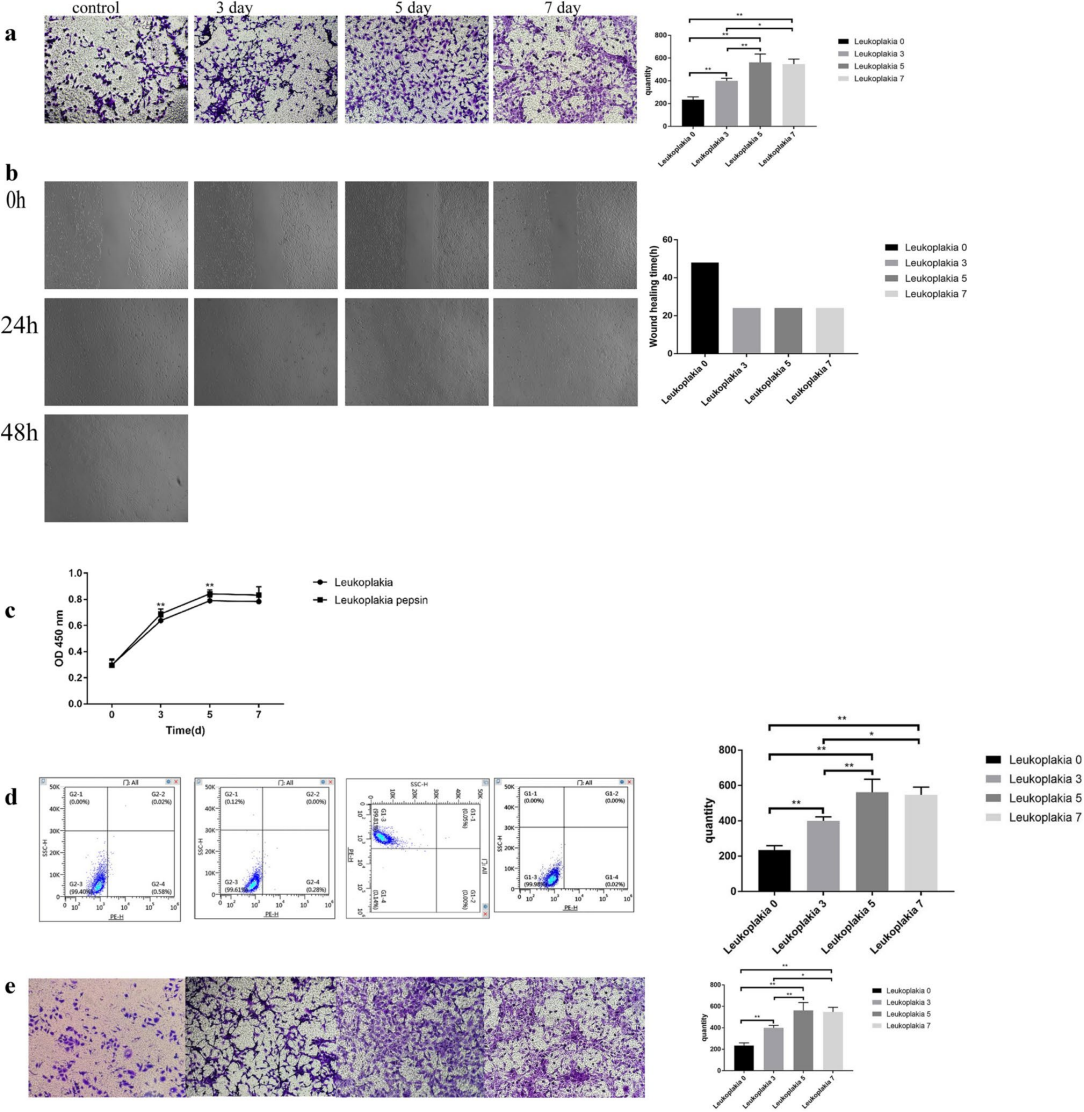
Migration, proliferation, apoptosis, and cell-cycle distribution of HVCLCs exposed to pepsin-containing artificial gastric juice. **a** Transwell assay; **b** wound-healing assay; **c** CCK-8 assay; **d** apoptosis; **e** cell-cycle distribution

**Fig. 3:**
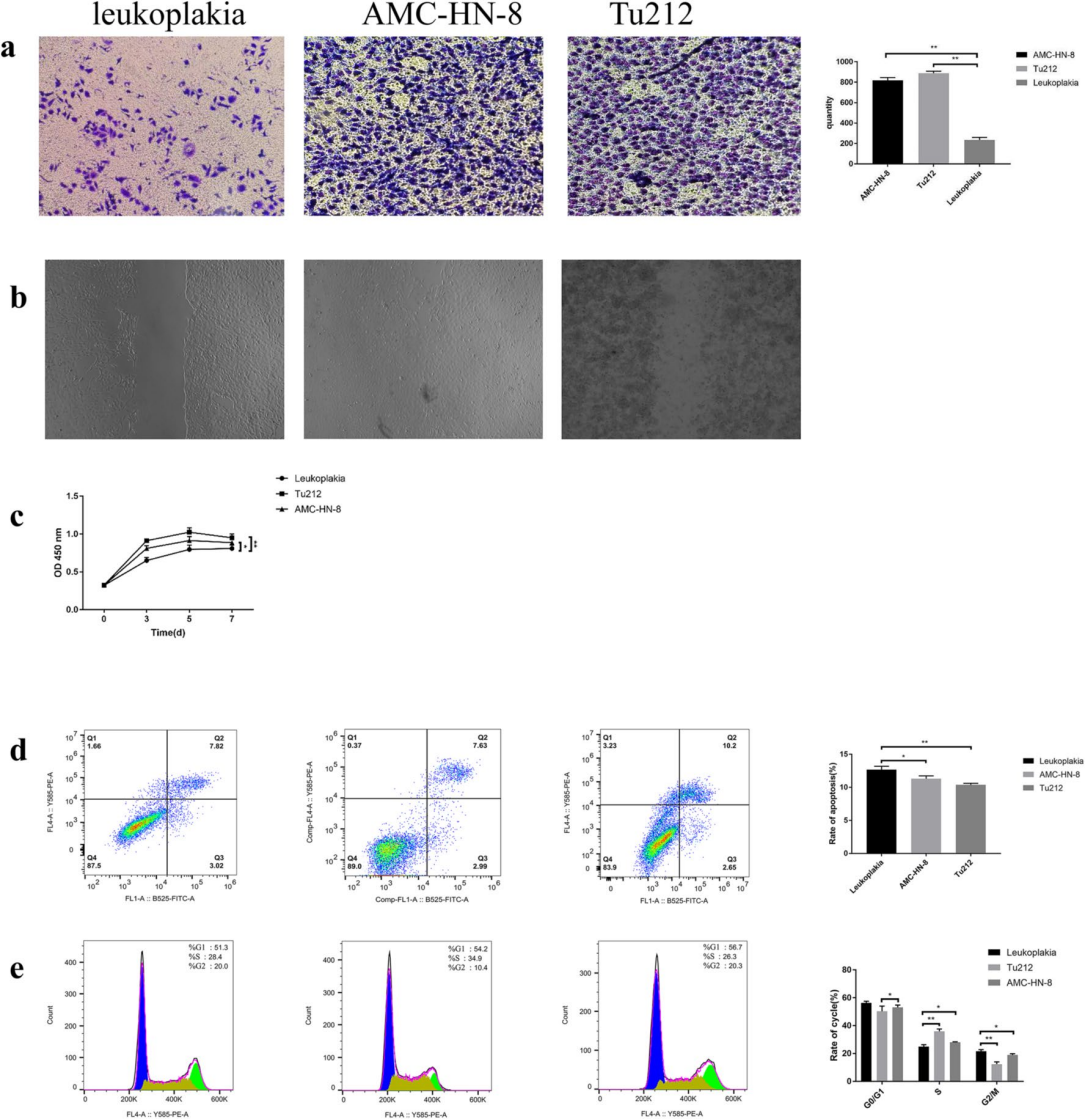
Compare to laryngeal carcinoma cells, migration, proliferation, apoptosis, and cell-cycle distribution of HVCLCs exposed to pepsin-containing artificial gastric juice. **a** Transwell assay; **b** wound-healing assay; **c** CCK-8 assay; **d** apoptosis; **e** cell-cycle distribution

Exposure to pepsin-containing artificial gastric juice significantly enhanced the migration and proliferation of VSCLCs in a time-dependent manner (p < 0.05, Fig. 2a–c), with a peak on day 5. However, the migration of VSCLCs was less obvious than that of laryngeal carcinoma AMC-HN-8 cells and TU212 cells (*p* < 0.05; Fig. 3a–c). The apoptotic rate of VSCLCs was higher than that of laryngeal carcinoma AMC-HN-8 cells and TU212 cells (*p* < 0.05, Fig. 3d). The apoptotic rate of VSCLCs decreased over time after exposure to pepsin and reached a nadir on day 7 (*p* < 0.01, Fig. 2d). The proportion of VSCLCs in S phase was significantly increased compared to laryngeal carcinoma AMC-HN-8 cells and TU212 cells (*p* < 0.05, Fig. 3e). With increasing duration of exposure to pepsin, the proportion of VSCLCs in G0/G1 phase decreased and the proportions in the S and G2/M phases significantly increased (*p* < 0.05, Fig. 2e). Therefore, pepsin promoted the migration, and proliferation of VSCLCs, but inhibited their apoptosis.

**Fig. 4:**
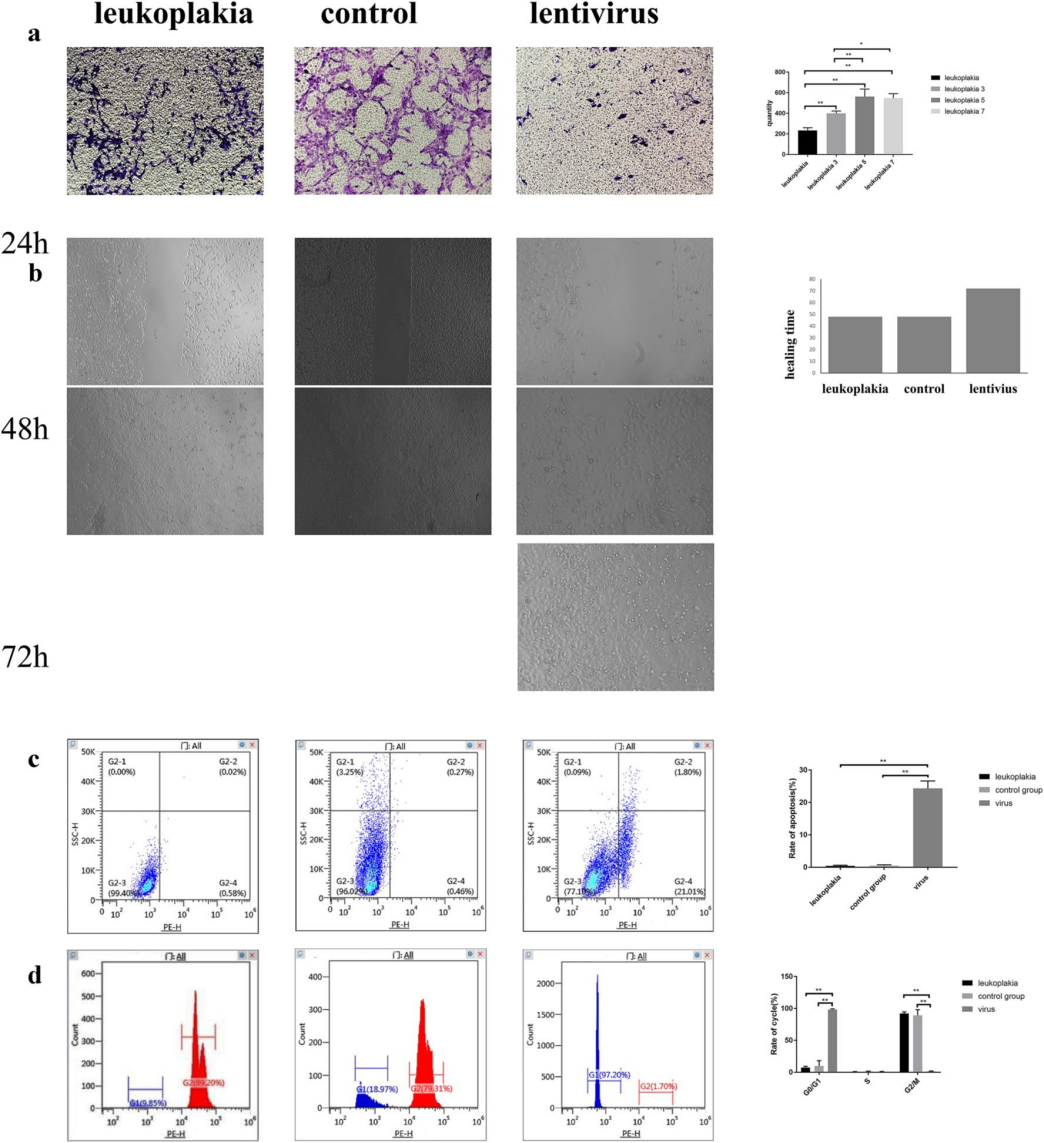
Migration, proliferation, apoptosis, and cell-cycle distribution of HVCLCs after transfection with Glut-1 low-expressing lentivirus. **a** Transwell assay; **b** wound-healing assay; **c** CCK-8 assay; **d** apoptosis; **e** cell-cycle distribution

Lentivirus-mediated inhibition of Glut-1 expression in VCL significantly inhibited the cells’ migration and proliferation (*p* < 0.05) but enhanced their apoptosis (*p* < 0.05). Also, inhibition of Glut-1 expression resulted in an increased proportion of cells in G0/G1 phase and a significantly decreased proportion in G2/M phase (*p* < 0.05, Fig. 4).

### Glut-1 and H^+^/K^+^-ATPase α, β expression

In 2D and 3D models, Glut-1and H^+^/K^+^-ATPase α, β expression was significantly lower in VCL cells than in Tu212 laryngeal carcinoma cells and AMC-HN-8 cells (*p* < 0.05, Figure.S1). Also, Glut-1 and H^+^/K^+^-ATPase α, β expression in VCL, Tu212 laryngeal carcinoma cells and AMC-HN-8 cells was positively correlated. Therefore, Glut-1 modulates the physiological characteristics of cells in precancerous lesions.

**Fig. 5:**
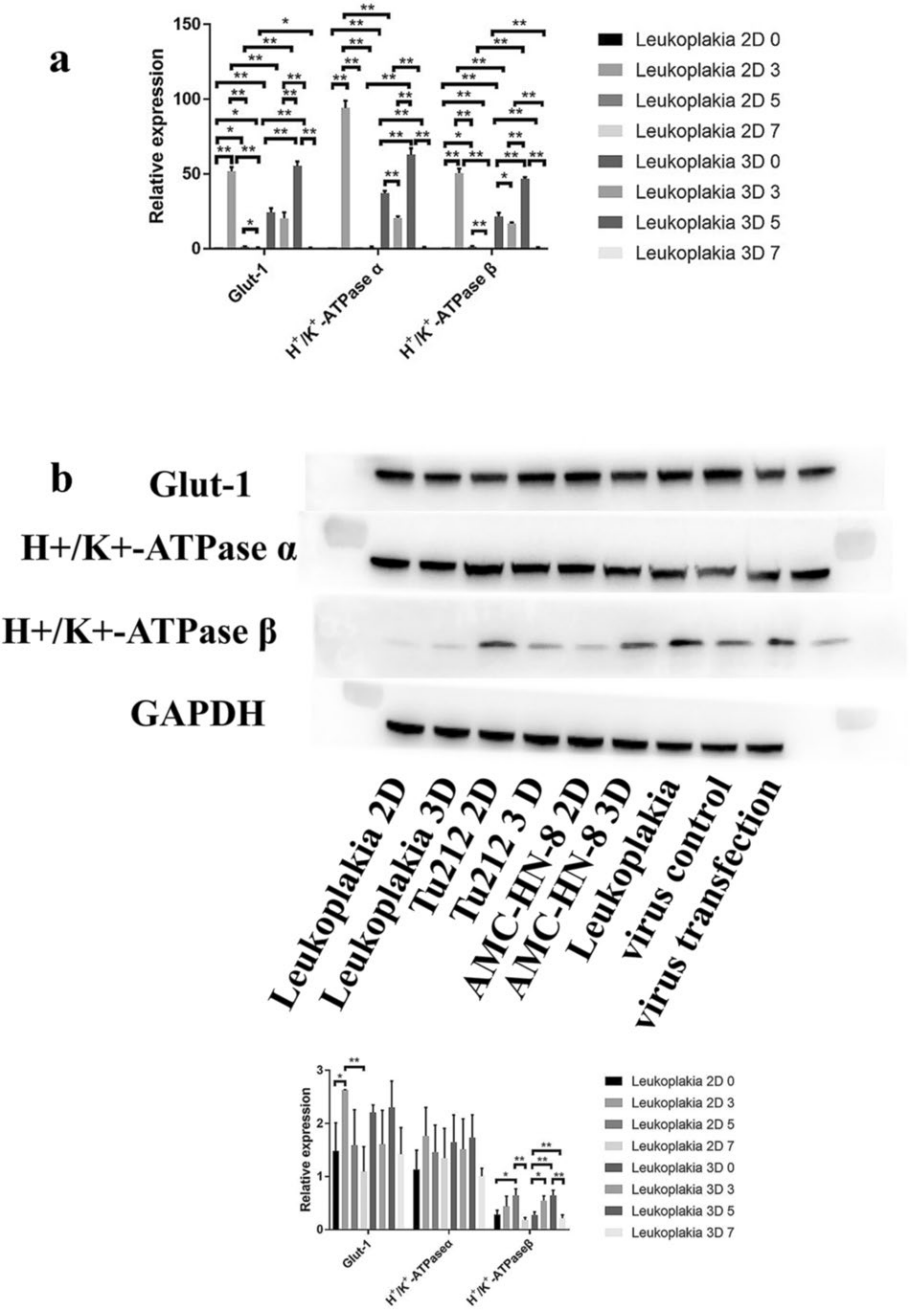
Glut-1 and H^+^/K^+^-ATPase α, β expression in 2D and 3D models of HVCLCs exposed to pepsin-containing artificial gastric juice by **a** RT-PCR and **b** Western blotting

In 2D and 3D models of VCL, pepsin-containing artificial gastric juice significantly increased Glut-1 and H^+^/K^+^-ATPase α, β expression compared to that in the absence of pepsin (*p* < 0.05, Fig. 5).

Inhibition of Glut-1 expression in VCL significantly decreased Glut-1 and H^+^/K^+^-ATPase α, β expression compared to both control groups (*p* < 0.01, Figure.S2). Moreover, Glut-1 expression was positively correlated with that of H^+^/K^+^-ATPase α, β. Western blotting showed that Glut-1 low-expression lentivirus inhibited the expression of Glut-1 protein only after transfection (*p* < 0.01, Figure.S2), albeit not significantly. Therefore, transfection with Glut-1 low-expressing lentivirus affected H^+^/K^+^-ATPase α, β expression only at the transcriptional level.

### Glut-1 and H^+^/K^+^-ATPase α, β expression in laryngeal carcinoma and vocal cord leukoplakia tissues

Glut-1 and H^+^/K^+^-ATPase α, β expression in laryngeal carcinoma tissues was higher than that in leukoplakia tissues (*p* < 0.05, Fig. S3a), which was higher than their expression in paracancerous tissue (*p* < 0.05, Fig. S3). IHC showed a similar tendency; however, only Glut-1 expression differed significantly between laryngeal carcinoma and leukoplakia tissues, and between leukoplakia tissues and paracancerous tissues (*p* < 0.05, Fig.S3).

### Associations of Glut-1 and H^+^/K^+^-ATPase α,β expression with clinicopathological features

Glut-1 expression was significantly higher in early stage (T_1_ + T_2_) than in advanced stage (T_3_ + T_4_) laryngeal carcinoma (*p* < 0.05). Also, expression of H^+^/K^+^-ATPase β was lower in well-differentiated laryngeal carcinoma than in moderately differentiated laryngeal carcinoma (*p* < 0.05). IHC showed that in patients with leukoplakia, H^+^/K^+^-ATPase α and β expression were higher in those ≥ 60 years old than in those < 60 years old (*p* < 0.05). There was no significant correlation between Glut-1 and H^+^/K^+^-ATPase α, β expression with any other clinicopathological feature of patients with laryngeal carcinoma or leukoplakia.

## Discussion

Glut-1 modulates the Warburg effect by regulating glucose uptake into tumor tissues. We have reported that expression of Glut-1 in laryngeal carcinoma is elevated [27] and was associated with the proliferation and radioresistance of laryngeal carcinoma cells; targeted inhibition of Glut-1 expression suppressed their proliferation and radioresistance [26–28]. Although expression of Glut-1 was lower in HVCLCs than in laryngeal carcinoma Tu212 and AMC-HN-8 cells, we detected Glut-1 expression in laryngeal precancerous lesions. The expression level of Glut-1 was associated with the migration, proliferation, apoptosis, and cell-cycle distribution of HVCLCs. Inhibition of Glut-1 expression decreased the migration and proliferation, and increased the apoptosis, of HVCLCs, as well as increasing the proportion in G0/G1 phase and decreasing that in G2/M phase. Therefore, Glut-1 alters the physiological properties of cells of laryngeal precancerous lesions. Others have shown that Glut-1 expression is upregulated in premalignant lesions (endometrial hyperplasia, premalignant lesions of the gallbladder, and oral leukoplakia and epithelial dysplasia) [29–32].

The mechanisms underlying the formation and development of VCL are unclear. LPR is associated with laryngeal precancerous lesions [5, 10], and pepsin is implicated in their development. We have reported that 68.0% of 45 pathologic specimens from patients with VCL were positive for pepsin, and pepsin expression increased as the grade of dysplasia of VCL increased1. H + /K + -ATPases are expressed in the larynx, as in the stomach [18, 20, 21]. Also, we detected H + /K + -ATPase α, β expression in normal laryngeal tissues by RT-PCR, Western blotting, and IHC. The distribution and expression level of H + /K + -ATPase α did not differ significantly among the various laryngeal subregions. However, the expression level of the β-subunit was higher in the epiglottic cartilage based on Western blotting [19]. Laryngeal H + /K + -ATPases may alter seromucinous secretion and regulate the acidic laryngeal microenvironment [18, 20, 21]. Therefore, acid secreted by laryngeal H + /K + ATPases may reactivate pepsin absorbed in the laryngeal epithelium, leading to damage to the laryngeal mucosa, laryngitis, and tumorigenesis. In this study, exposure to pepsin-containing artificial gastric juice enhanced the invasive and migratory ability of HVCLCs and increased and decreased the proportions in the S and G2/M phase and G0/G1 phase, respectively. Therefore, pepsin is implicated in the development of VCL. After exposure to pepsin-containing artificial gastric juice, Glut-1 and H + /K + -ATPase α, β expression was correlated with the migration, proliferation, cell-cycle distribution, and apoptosis of HVCLCs. Also, inhibition of its expression indicated that Glut-1 regulates the expression of H + /K + -ATPase α, β in HVCLCs. Our findings indicate that pepsin plays a role in the development of VCL by increasing Glut-1 expression, causing elevated expression of H + /K + -ATPase α, β in HVCLCs. Pepsin damages the mitochondria of laryngeal epithelial cells, causing reprogramming of glucose metabolism [13, 16]. High expression of Glut-1 elevated glycolysis, leading to increased lactate, reducing the microenvironment pH and thus reactivating pepsin.

Also, Glut-1 and H + /K + -ATPase α,β expression in 30 laryngeal carcinoma adjacent tissues, 30 VCL tissues, and 30 laryngeal carcinoma tissues increased gradually (*p* < 0.05). Glut-1 expression was correlated with H + /K + -ATPase α, β expression in HVCLCs. We reported previously that H + /K + -ATPase α, β expression increased in order in normal laryngeal tissues (30 specimens), paracarcinoma tissues (30 specimens), and laryngeal carcinoma tissues (30 specimens) by RT-PCR, Western blotting, and IHC. H + /K + -ATPase α, β expression were significantly higher in laryngeal carcinoma than in paracarcinoma tissues. McCormick et al. reported that H + /K + -ATPase α (ATP4A) and β (ATP4A) were expressed in laryngeal carcinoma tissues, paracarcinoma tissues, and laryngeal tissues from patients with LPR. However, there was no significant difference in expression, likely because of the small number of samples [16]. Therefore, H + /K + -ATPase is expressed in normal laryngeal tissues and is elevated during laryngeal epithelial hyperplasia and malignant transformation. However, the role of Glut-1 and H + /K + -ATPase α, β expression in the pathogenesis of laryngeal carcinoma is unclear. Therefore, large prospective, multicenter studies are needed to clarify the mechanism of Glut-1-mediated H + /K + -ATPase expression in laryngeal carcinogenesis, and the role of pepsin in this process.

This study had several limitations. First, we did not investigate the mechanism by which pepsin induces laryngeal malignant transformation. Second, the differences between 3D- and 2D-cultured cells should be explored. Finally, this was a retrospective study, and we did not apply the 24 MII-pH, reflux symptom index, or reflux finding score.

## Conclusions

Pepsin-containing artificial gastric juice promoted the invasion and migration of HVCLCs. Pepsin-induced development of precancerous lesions is associated with abnormal expression of Glut-1 and H^+^/K^+^-ATPase α, β. The expression of Glut-1 and H^+^/K^+^-ATPase α, β gradually increases from laryngeal precancerous lesions to laryngeal carcinoma. Therefore, Glut-1 may mediate the development of laryngeal precancerous lesions by upregulating H^+^/K^+^-ATPase expression, reactivating absorbed pepsin and damaging the laryngeal mucosa.

## Supplementary Information

Below is the link to the electronic supplementary material.

Supplementary file1 (TIF 7683 KB) Glut-1 and H+/K+-ATPase α,β expression in 2D and 3D models with HVCLCs, Tu212 laryngeal carcinoma cells and AMC-HN-8 cells by (a) RT-PCR and (b) Western blotting

Supplementary file2 (TIF 43348 KB) Glut-1 and H+/K+-ATPase α, β expression in HVCLCs transfected with Glut-1 low-expressing lentivirus by a. RT-PCR and b. Western blotting

Supplementary file3 (TIF 84342 KB) Glut-1 and H+/K+-ATPase α,β expression in laryngeal carcinoma and VCL tissues by RT-PCR (a), Western blotting (b), and IHC (c)

## Data Availability

Data are available on request to the authors.
