# Supplementary figures and images for: Role and mechanism of Glut-1 and H+/K+-ATPase expression in pepsin-induced development of vocal cord leukoplakia
Source: Eur Arch Otorhinolaryngol. 2021 Nov 20;279(3):1413–24. doi: 10.1007/s00405-021-07172-y (PMC8897356; doi:10.1007/s00405-021-07172-y)

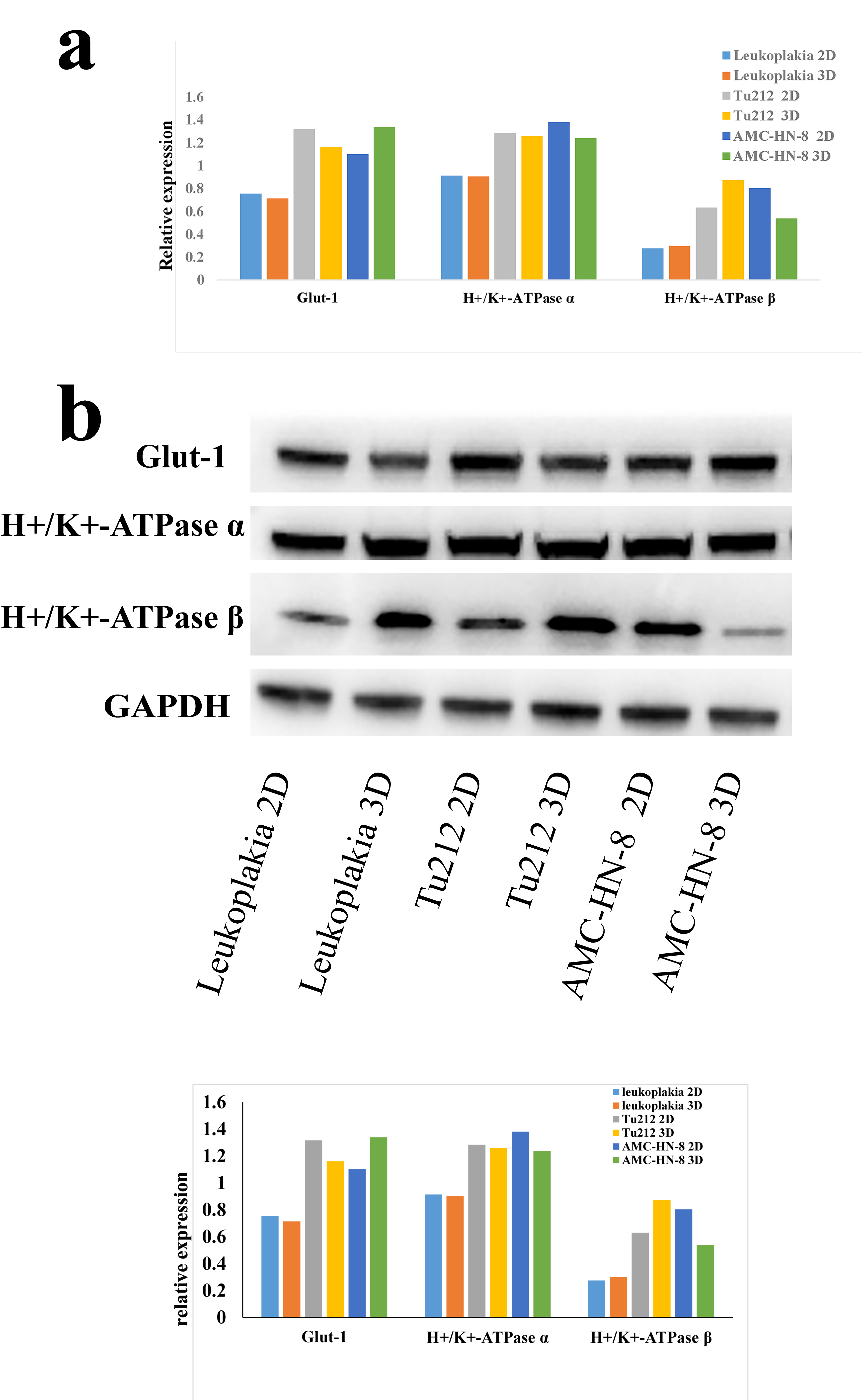

Supplement: Supplementary file 1 — Supplementary file1 (TIF 7683 KB) Glut-1 and H+/K+-ATPase α,β expression in 2D and 3D models with HVCLCs, Tu212 laryngeal carcinoma cells and AMC-HN-8 cells by (a) RT-PCR and (b) Western blotting [file 405_2021_7172_MOESM1_ESM.tif]

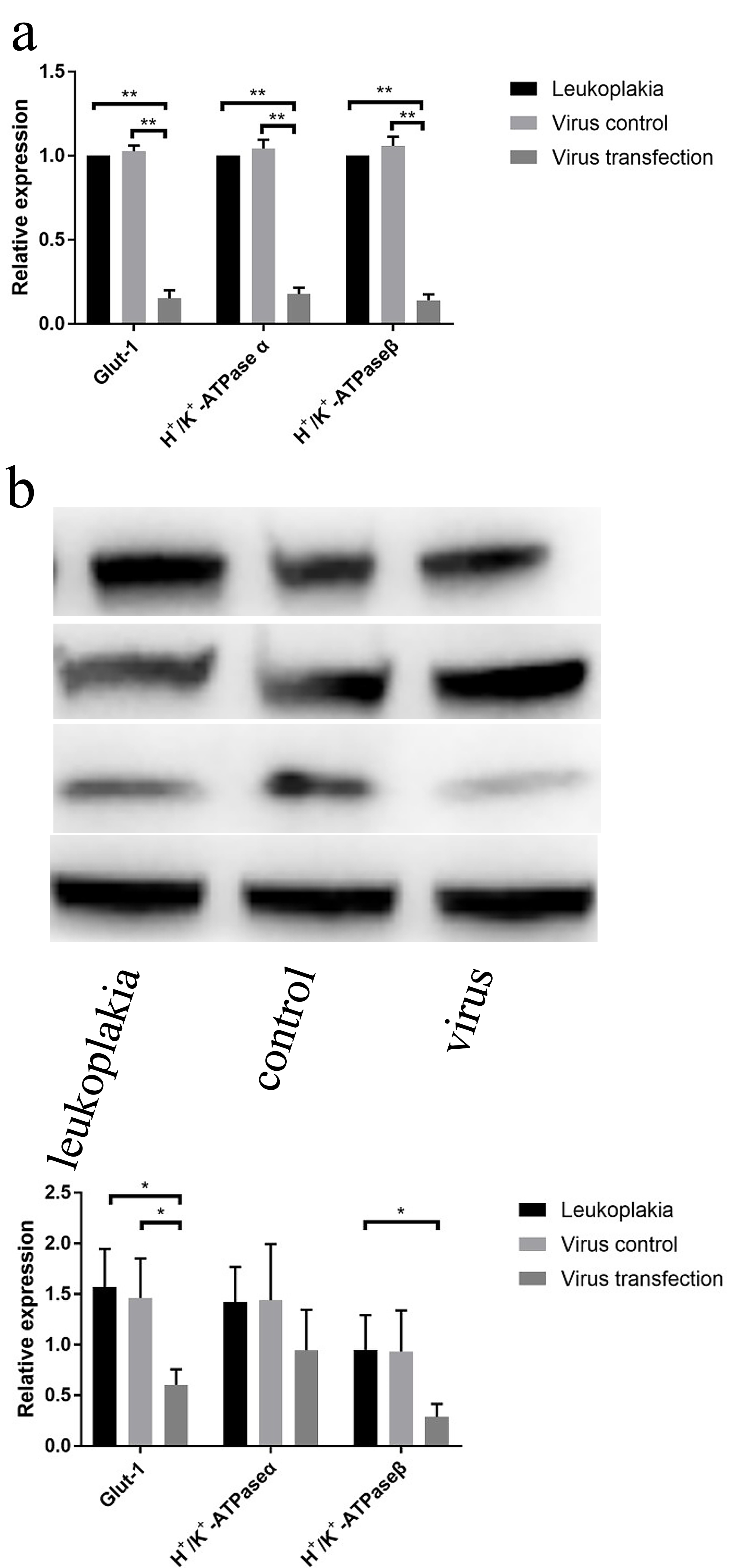

Supplement: Supplementary file 2 — Supplementary file2 (TIF 43348 KB) Glut-1 and H+/K+-ATPase α, β expression in HVCLCs transfected with Glut-1 low-expressing lentivirus by a. RT-PCR and b. Western blotting [file 405_2021_7172_MOESM2_ESM.tif]
